# The effects of live streamer’s expertise and entertainment on the viewers’ purchase and follow intentions

**DOI:** 10.3389/fpsyg.2024.1383736

**Published:** 2024-03-20

**Authors:** Yaping Jiang, Hyoung-Tark Lee, Wei Li

**Affiliations:** ^1^School of Business, Shanghai Normal University Tianhua College, Shanghai, China; ^2^School of Business, Keimyung University, Daegu, Republic of Korea; ^3^School of Economics and Management, East China Normal University, Shanghai, China

**Keywords:** streamer characteristics, trust, flow experience, purchase intention, follow intention, optimal stimulation level

## Abstract

This study explores the impact of two characteristics of streamers—expertise and entertainment—on viewers’ purchase intention and follow intention in live-streaming e-commerce, with a specific focus on viewers’ trust and flow experience as two mediators and viewers’ optimal stimulation level as a moderator. We implemented a methodological approach where participants were randomly directed to enter a live broadcast room and watch a 10-min live session before engaging in a structured questionnaire. 399 valid questionnaires were collected from the participants. These 399 valid questionnaires were subsequently utilized to validate the research model using structural equation modeling (SEM). The results suggest that streamer expertise and entertainment enhance viewers’ trust and flow experience, which then leads to an increase in their intention to make a purchase and continue following the streamer. Furthermore, the viewers’ optimal stimulation level acts as a moderator in the connections between streamer characteristics and viewers’ trust and flow experience, suggesting that individual differences among consumers affect how they respond to streamer characteristics. From the dual perspectives of the streamer and the viewer, this study provides a more comprehensive theoretical perspective on customer behavior in live streaming commerce by not only focusing on consumers’ short-term, transactional behavior inclinations but also long-term, relational behavior intentions.

## 1 Introduction

The live streaming commerce business in China has significantly grown due to the progress of emerging social media platforms. According to industry reports (Wisdom Consulting, 2023), the market size of China’s live streaming commerce in 2022 amounted to $51.86 billion, which is almost 178 times more than the 2.91 billion dollars recorded in 2017. The total count of live streaming sessions on prominent e-commerce platforms surpassed 120 million, with a fivefold increase compared to the previous 2 years. The transaction volume had a significant increase from 3.98 billion dollars in 2017 to 368.96 billion dollars in 2022, demonstrating a compound annual growth rate of 147.4%. The quantity of live-streaming e-commerce businesses rose from 2,915 in 2017 to 18,700 in 2022, exhibiting a compound annual growth rate of 45.02%. The online live streaming user base in 2022 reached a total of 751 million. Among these users, 473 million engaged in live streaming commerce, making up 63% of the whole online live-streaming audience and 44.3% of the entire internet population.

The increasing ubiquity of live streaming commerce has attracted considerable attention, especially from certain researchers. Their primary emphasis was on the following five perspectives: (1) Considering the streamer attributes, scholars have adopted different dimensions to categorize streamer characteristics and explored the mechanisms of influence on consumer behavior (Guo et al., 2022; Rungruangjit, 2022; Xu et al., 2022; Yang et al., 2023; Zheng et al., 2023). (2) From a consumer viewpoint, some scholars have examined how consumer-streamer resonance impacts parasocial interaction, engagement, and subsequently, purchase intentions (e.g., Shen et al., 2022). (3) From the perspective of the product, studies have explored the effects of anchor-, live content-, Danmaku content-, and self-product fit on consumers’ purchase intentions by reducing different types of product uncertainty (Park and Lin, 2020; Chen et al., 2023). (4) From the perspective of the live broadcasting environment, scholars have examined how visual complexity affects consumers’ emotions and subsequently influences their purchase intentions (Tong et al., 2022), as well as the impact of background music placement on consumers’ emotions and consumer memory, thereby influencing purchase intentions (Zhang et al., 2023). (5) From the perspective of the media and technological attributes, studies have explored how media characteristics of the live streaming platform, such as perceived interaction control, human-message interaction and perceived invasiveness influence consumer attitudes and purchase intentions (e.g., Bawack et al., 2023). Draw from literature review, it can be found that the studies on consumer behavior in live streaming e-commerce predominantly focuses on consumers’ purchase intentions (e.g., Clement et al., 2021; Shen et al., 2022; Zheng et al., 2022; Vazquez et al., 2023), reflecting short-term, transactional behavior responses. These studies are centered on how live e-commerce platforms can facilitate immediate sales and market conversions. However, a significant gap remains in the research regarding long-term, relational behavior responses, specifically, consumers’ sustained attention to streamers (follow intention).

With the fast-developing live commerce, streamer characteristics are becoming an essential part of drawing consumer engagement and boosting sales transformation ratios (Meng et al., 2021; Guo et al., 2022; Chen et al., 2023; Liu et al., 2023; Ma et al., 2023; Yang et al., 2023). The expertise of streamers is viewed as a reflection of their credibility and trustworthiness (Zheng et al., 2022), which matters in the virtual marketplace sector. Consumers usually look for authoritative voices that can offer accurate information and product guidance. Not only is expertise an indicator of the streamer’s level of knowledge but also a symbol that he or she can pass on information in an effortless way and recommend stuff confidently, definitively. It can provide reassurance, foster trust, and have a significant impact on their behavioral intentions (Zhou and Tong, 2022). On the other hand, the entertainment aspect fulfills the consumers’ need for enjoyment (Weiwei and Yongyue, 2021) and captures their attention (Yin et al., 2024), making it another essential characteristic. In the highly competitive live streaming environment, the ability to entertain is key to maintaining consumer engagement, creating memorable experiences, and building emotional connections with the audience. This entertainment aspect increases consumer retention and can transform passive viewers into active participants, and eventually into customers. Considering live streaming as both a marketing tool and an entertainment medium, this study focuses on the expertise and entertainment provided by streamers, exploring how these traits influence consumer behavior.

Consumer decision-making processes are not unidimensional but are influenced by multiple factors. In virtual environments, consumers are unable to directly touch and assess products. Therefore, they often rely on the credibility of the streamers as a source of information. Perceived trust not only facilitates short-term transactions (Zhou and Tong, 2022) but also contributes to the establishment of long-term customer relationships. Flow experience describes an individual’s complete immersion and high concentration state during an activity (Kim and Kim, 2022). In live commerce environments, when consumers experience flow, they are more likely to engage in live streams for extended periods, increasing the likelihood of interacting with content and making purchases (Hyun et al., 2022). Based on this, this paper introduces trust and flow experience as key variables influencing consumer behavior toward streamers. Additionally, this paper also introduces the optimal stimulation level, which reflects individuals’ ideal level of stimulation from the environment (Helm and Landschulze, 2009), to explore how consumer intrinsic factors influence their reactions during live streaming.

To summarize, this study poses three research questions: (1) Do the streamers’ characteristics, e.g., expertise and entertainment abilities affect consumers’ purchase intentions as well as their desire to follow a specific steamer? (2) If the influence is present, what are its mechanisms? (3) Do personal characteristics of consumers (optimum stimulation level) play a moderating role in this process? To gain a comprehensive understanding of the potentially rapidly emerging live streaming commerce market globally, this study focuses on analyzing customers active in the live streaming commerce sector in China, aiming to understand the unique characteristics of live streaming commerce. This study will adopt a long-term perspective, introducing the relational variable of ‘follow intention’. Considering the critical role of streamer characteristics in live streaming commerce, this study will explore how the expertise and entertainment of streamers affect both purchase and follow intentions, incorporating consumer trust and the experience of flow as mediating variables, and considering the optimal stimulation level of consumers as a moderating variable.

## 2 Literature review

### 2.1 Streamer characteristics

Drawing on the insights from source credibility theory and uses and gratifications theory, our study examines the effects of streamer characteristics through two key aspects: (1) a factor of expertise, in which the streamer’s competency appears as an expert; (2) The entertainment factor, is defined using its high level of interaction and entertaining style.

#### 2.1.1 Expertise

In most cases, expertise refers to the level at which a communicator (in this case, a streamer) is believed to be capable of supplying accurate, reliable, and correct details. The source credibility theory, which was first proposed by Hovland et al. (1953), takes expertise to be a major determinant of a sources persuasive power. A source’s expertise plays a significant role in the creation of beliefs, attitudes, and behaviors (Ohanian, 1991; Ladhari et al., 2020). If we consider the live-streaming context, a streamer’s expertise can have tremendous effects on the level of credibility that they possess which will eventually determine their powers to convince viewers. Previous research has demonstrated that it has a high correlation with attitudes, engagement, and trust, as well as consumer behaviors such as purchase intention (e.g., Ismagilova et al., 2020; Zheng et al., 2023; Li et al., 2024).

#### 2.1.2 Entertainment

According to the theory of Uses and Gratifications (Katz et al., 1973), people actively seek media channels that can fulfil particular needs or gratify them such as entertainment. When considering live streaming, the streamers often present entertainment that assists in viewer’s enjoyment or relaxation goals and escapism. Research on social media and influencer marketing shows that entertainment is a crucial component of viewer engagement, content sharing, as well loyalty by followers (De Veirman et al., 2017; Matthes et al., 2023). In live streaming, the entertainment factor is often associated with viewers’ flow experience, perceived value, trust, attitude, and purchase intention (e.g., Chen and Lin, 2018; Liu et al., 2022; Ong et al., 2024).

### 2.2 Purchase intention

There is an abundance of existing research on purchase intention. These studies collectively suggest that a range of factors, including corporate social responsibility (e.g., David et al., 2005), human interaction (e.g., Hu et al., 2016; Yin et al., 2019), product type (e.g., Moon et al., 2008; Hsu et al., 2017), online search behavior (e.g., Shim et al., 2001), and key technical aspects of a social shopping website (e.g., Hsu et al., 2012; Hu et al., 2016), can significantly influence purchase intention. In live streaming commerce context, consumers’ purchase intention is influenced by a variety of factors, including factors related to the streamer, IT factors, product-related factors, platform-related factors, and consumer-related factors (Gao et al., 2023). For instance, Zhang et al. (2023) found that the placement of background music in live streaming commerce significantly impacts consumers’ arousal, memory, and purchase intention. Tong et al. (2022) demonstrated that background visual complexity positively influences purchase intention by stimulating consumers’ emotional states. Shen et al. (2022) highlighted that when a viewer’s self-image and values fit with those of a streamer, it positively influences their desire to make a purchase. Draw from e-tailers’ perspective, Zheng et al. (2022) established a clear association between customer interaction activities and both purchase intention and customer acquisition. Although factors influencing purchase intentions have been extensively studied in the real-time commerce field, certain aspects remain unclear. For instance, the influence of streamer attributes on viewers’ trust, flow experience, and subsequent purchase intentions, is still not fully understood. In addition, past studies have usually focused on one aspect only, and there have been few studies from the dual perspectives of streamer characteristics and consumer characteristics. This research aims at filling these gaps.

### 2.3 Follow intention

The term “follow intention,” which is the creation of this research, bases on viewers’ tendency to keep following and interacting with streamers in response to such factors as those that are peculiar for a certain streamer (e.g., characteristics), consumer trust, or flow experience enjoyed by them. “Follow intention” shares similarities with the “subscribe intention” variable proposed by previous scholars in the study of traditional e-commerce, video and audio streaming platforms (such as YouTube, Netflix), blogs, and similar platforms. The reason for introducing the “follow intention” variable is based on its greater relevance to live streaming scenarios. Numerous studies point to the significance of influencer attributes and digital marketing strategies in defining viewer behaviors and decisions. For instance, Nagaraj et al. (2021) sought to illustrate that Indian consumers usually consider such factors as content, convenience, price, and quality when shaping their inclination to subscribe for OTT (Over-The-Top) video streaming services. The factors of perceived ease of use, security, sovereignty structure to ensure trust, and system quality satisfaction have an impact on the intention of emerging economies to continue using platform-based governance services (Upadhyay et al., 2022). As stated by Horng et al. (2016) and Pereira and Tam (2021), the framework of the long-term relationship between audience loyalty and digital content reveals the role of trust perception embedded in user satisfaction, which comes from watching various videos produced by different authors (technology bloggers or hardware manufacturers). Recommendations or suggestions from the content creators themselves directly impact on a user making decisions upon which subsequent actions they make after viewing their video for example subscribing and even purchasing via recommendation (Esposito et al., 2015; Chakraborty et al., 2023). These insights are closely related to the subject of this study - follow intention, which is reflects the consumer’s interest and engagement with specific live streamers and is a core indicator in the live streaming commerce field. It helps platforms and streamers understand their audience and measure the appeal and influence of their content, thereby guiding the development of effective marketing strategies and content creation plans. Hence, there is a clear need to combine the perspectives of short-term transactional variables (purchase intention) and long-term relational variables (follow intention) to gain a more comprehensive understanding and insight.

### 2.4 Flow experience

The concept of flow experience was initially described by Csikszentmihalyi (1975). It is called flow; the condition, in which a person finds oneself while fully immersed and focusing on an object or activity with all attention span that he has belonging to his own stream of consciousness deeply resonating with this content. Various studies, for instance, Zhang et al. (2023), Sun et al. (2019), and Liu et al. (2022) have pointed out that consumers’ flow experience plays a significant role in their purchase decisions during live streams. A state of engagement not only stimulates those clients toward current purchase but also serves as directed to consumer loyalty and retention, which is proved in the study by Kim and Kim (2022). Reflecting the importance of flow experience, this study reveals it as one of mediating variables to see how streamer expertise and entertainment would affect viewers’ follow intention and purchase intention.

### 2.5 Trust

Trust is the desire of an individual to believe in and rely on the reliability and integrity of an exchange partner (Morgan and Hunt, 1994; Sirdeshmukh et al., 2002). It encompasses the trust that can be assumed in digital contexts regarding, for instance, accuracy of information provided to security of transactions and assumptions about whether involved parties will act in good faith. Trust has been extensively researched and acknowledged as a key component in customer-seller relationships. For instance, Mayer et al. (1995) have pointed out that trust plays a significant role in the creation of effective relationships with consumers for digital platforms or service providers. Delgado-Ballester and Munuera-Alemán (2001) emphasized the central role of brand trust in the formation of consumer loyalty and commitment, particularly in contexts of high engagement. According to Garbarino and Johnson (1999), trust, which refers to how reliable and honest a transaction partner is perceived to be, exerts a substantial impact on client perceptions and subsequent intentions. In live streaming commerce, scholars have found that a lot of trust-related behaviors are influenced by certain aspects such as the competence of streamers, mode and authenticity in communication (Wongkitrungrueng and Assarut, 2020; Guo et al., 2021; Liu et al., 2022; Ma et al., 2022; Zhou and Tong, 2022). Therefore, this study proposes to explore how trust serves as a crucial intermediary, potentially affecting how the attributes of streamers, such as their knowledge and ability to entertain, influence consumer behavior in terms of their willingness to follow and make purchases.

### 2.6 Optimal stimulation level

Optimal stimulation level (hereinafter referred to as OSL) describes how much mental or sensory activity an individual wants to engage in Mittelstaedt et al. (1976). The finding of (Zheng et al., 2023) consists in that persons scoring highly on OSL prefer higher levels of stimulation for hedonic reasons and are novelty oriented, while those with low scores enjoy being in familiar environments. OSL significantly influences consumer behavior and preferences (Helm and Landschulze, 2009), such as their interactions with digital content (Lichtlé, 2007) or online shopping (Richard and Chebat, 2016). People with higher OSL are more likely to make full use of the content and therefore they will probably have a greater engagement in relation to engaging, diverse, and new streaming content, while people with lower OSL may want clear structure as well as all the usual nuances that psychological operations might consider differently. Given the vital nature of OSL that shapes users’ sentiments on reactions assigned to streamed content, this study acknowledges it as a significant moderating variable in building up viewers’ trust and flow experience.

## 3 Research model and hypothesis development

### 3.1 The effect of streamer characteristics

#### 3.1.1 The effect of streamer expertise

A set of information that can be found in the stream is related to expertise and skillfulness in a particular sphere, known as streamer expertise (Zhang et al., 2022a). As Zhu et al. (2021) note, in this knowledge input, the aspect of consumer behavior and trust is strategically defined from an expert’s perspective. The fact that the streamers display specialized knowledge in a given field increases a consumer’s likelihood of considering them as credible thus believing any product endorsement made by the streamer. Such a connection leads to a favorable perception of the products endorsed (Zhang et al., 2021), thereby reinforcing consumers’ trust (Li et al., 2024).

Additionally, the deep level of knowledge and skill provided by the streamer leads to an immersive and engaging viewer experience (Li and Peng, 2021), where consumers are more absorbed and involved in the content. The proficiency of streamers results in increased audience engagement, so enhancing the immersive experience of live streaming and fostering a heightened sensation of flow (Kim and Kim, 2022; Liao et al., 2022). Consequently, the study puts forward the following hypotheses:

*H1a:* Streamer expertise positively influences viewers’ trust.

*H1b:* Streamer expertise positively influences viewers’ flow experience.

#### 3.1.2 The effect of streamer entertainment

Entertainment refers to activities designed to captivate and hold the audience’s attention, aiming to bring joy, spark interest, or provide a respite from daily life by offering a momentary diversion from reality (Chen and Lin, 2018). This allows the audience to set aside their concerns and immerse themselves in an enjoyable experience (Liu et al., 2022), which lead the audience to consume, create or contribute to online content related to a particular brand (Muntinga et al., 2011). In the use of social networks, entertainment is an important motivation, reflecting users’ desire for fun, escape from daily stress, and enjoyment of the entertaining experiences provided by social networks (Curras-Perez et al., 2014). Mull and Lee (2014) claimed that entertainment serves as a primary incentive for individuals to trust and utilize Pinterest. Gan and Wang (2015) discovered that the primary incentive for using Wechat is the pursuit of leisure activities, encompassing the need for entertainment, enjoyable downtime, and relaxation. In live streaming context, when streamers are entertaining, they create a more engaging and immersive environment, which enhances the viewer’s trust and promotes intense focus and full absorption in the content. Thus, the research posits the subsequent hypotheses:

*H1c:* The entertainment provided by streamers positively influences viewers’ trust.

*H1d:* The entertainment provided by streamers positively influences viewers’ flow experience.

### 3.2 The effect of viewers’ trust

Trust, being a cornerstone in relationship marketing (Morgan and Hunt, 1994), influences consumer behavior by fostering confidence in the trustworthiness of the streamer. According to prevailing studies, trust significantly contributes to consumers’ confidence and conviction in a company and its products, playing an influential role in determining their purchasing behavior. For example, Lu and Zhang (2020) proved that a high level of trust can significantly speed up transactions between the business entity and consumers. Zhou and Tong (2022) stated that “atmospheric in the live studio will influence consumers’ perception of emotional value and trust, which finally affect their purchase intentions.” In the same vein, Zhu et al. (2021) found that parasocial interaction is highly influenced by streamers’ attributes and therefore the qualities of charm may trigger development of affective trust in their prospects influencing consumers shopping intentions when it comes to adopting livestreams. Furthermore, emotional trust was found to contribute to the purchasing intention of customers while facing streaming experience (Meng et al., 2021). The role of perceived trustworthiness was also focused by Park and Lin (2020), especially in relation to celebrity endorsement present in live streaming for online shopping.

There are different aspects that play into the trust shown by consumers to a content creator. These factors may include authenticity, reliability, entertainment, and quality of the contents. This trust element is crucial in deciding if a consumer will be connected to the creator over time. Nagaraj et al. (2021) stated that a creator’s post authenticity and quality highly influence the viewer. In addition, Upadhyay et al. (2022) also talked about how digital marketing strategies can impact the consumer behavior such as their willingness to associate with brands or creators post-watching content. Horng et al.’s (2016) as well as Pereira and Tam’s (2021) research emphasized the need to generate viewer loyalty and consistent engagement that are directly related to trust, satisfaction, and perceived value. Most viewers tend to continue engaging because of trust foundations created by live streaming through authentic and reliable interactions (Zhang et al., 2022b), being one of the factors that leads them into following a streamer for future content.

Thus, this study proposes the following hypotheses.

*H2a:* Viewers’ trust positively affects their purchase intention.

*H2b:* Viewers’ trust positively affects their follow intention.

### 3.3 The effect of viewers’ flow experience

Being in a flow state implies being fully involved with live streaming materials which cause much interaction and make one understand better what things or services the streamer is promoting. This heightened engagement may result in a better evaluation of the featured products or services as well as a perception of them being more attractive and desirable. The consumers’ emotional perception concerning the value that they gain from products increase with increased flow experience (Zhang et al., 2023), and thus influences purchase and follow intention. Liu et al. (2022) demonstrated that the state of flow has a beneficial impact on the intention of consumers to make purchases. Zheng et al. (2023) demonstrated that the flow state has a substantial impact on the propensity to engage in continuous viewing and the likelihood of making a purchase. When consumers are fully engrossed and completely involved in the live streaming environment, they are more likely to develop a stronger intent to purchase the products or services being showcased (Richard and Chebat, 2016; Sun et al., 2019; Huang et al., 2022; Hyun et al., 2022; Liao et al., 2022; Saffanah et al., 2023). Moreover, researchers have demonstrated that streamer skills positively influence the viewers’ emotional engagement (flow experience) and satisfaction (psychological well-being), which in turn affects their commitment and loyalty toward the streamers (Kim and Kim, 2022).

Based on the findings mentioned above, the following hypotheses are proposed.

*H3a:* Viewers’ flow experience positively affects their purchase intention.

*H3b:* Viewers’ flow experience positively affects their follow intention.

### 3.4 The mediating effect of viewers’ trust

Viewers are more likely to trust and follow a streamer’s product recommendations when they perceive the streamer as knowledgeable, reliable, and competent, improving their faith in the authenticity and quality of the products promoted. As a result, viewers are more inclined to develop a positive attitude toward the products and express a higher intention to make a purchase. Yang (2021) used trust theory and reference group theory in their empirical research to establish that consumer trust mediates the impact of internet celebrity characteristics on brand attitude, employing a structural equation model for this demonstration. Chen et al. (2023) examined how livestreaming e-commerce streamers’ coping strategies (active or avoidance) after failures impact viewers’ word of mouth (WOM), highlighting the mediating role of cognitive and affective trust in this process. Singh and Sinha (2020) concluded that perceived trust acts as a significant mediator in the connection between various factors (like perceived usefulness, compatibility, awareness, cost, and customer value addition) and merchants’ intention to adopt mobile wallet technology. Zhang et al. (2021) found that the characteristics of live streaming, including visibility, interactivity, authenticity, as well as entertainment, not only motivate consumers’ purchase intentions but also enhance their perceptions, which includes perceived trust and usefulness, thereby partially mediating the effect on their behavioral intentions. Given the aforementioned facts, this study presents the hypotheses as follows.

*H4a:* Viewers’ trust mediates the effects of streamer expertise on purchase intention.

*H4b:* Viewers’ trust mediates the effects of streamer expertise on follow intention.

*H4c:* Viewers’ trust mediates the effects of streamer entertainment on purchase intention.

*H4d:* Viewers’ trust mediates the effects of streamer entertainment on follow intention.

### 3.5 The mediating effect of viewers’ flow experience

Streamers who are perceived as knowledgeable or skilled will very likely put viewers in a state of flow, which will increase their engagement with the content and thus will raise the possibility of converting them into those users that make purchases and follow streamers after watching. Moreover, flow can be made possible through entertaining aspects of content that may arrest viewer attention and help maintain their interest. If viewers like the content, they become involved more and may have interest to purchase its items as well as subscribe for watching the streamer. According to the findings of Liu et al. (2020), interactive techniques used by internet celebrities, such as real-time product demonstrations, detailed product introductions with precise operations, and the resulting emotional connection with customers, lead to the development of a feeling of being present among consumers. Subsequently, a flow experience is established, followed by an increase in purchase intention, leading to more items being added to the cart. Yu and Xu (2017) found that consumers’ flow experience based on immersion, distortion in terms of time feel experienced by a person, sense associated with control as well as curiosity for information has been observed more when the expertise shown from an info source are usually prominent. Zheng et al. (2023) asserted that the flow experience serves as a mediator in the relationship of different live streaming attributes, with continuous watching along with purchasing intentions by viewers. The conclusion of Chen and Lin (2018) stated that flow experience is a mediating factor in the relationships between entertainment, social interaction, endorsement and viewers’ intentions to use live streaming. In other words, these elements create more exciting stream contents which actually increase attention power on watching such streams through enhancing with favorite aspects like appealing acting style or any tragic climax etc. Therefore, this study suggests the following hypotheses.

*H5a:* Viewers’ flow experience mediates the effect of streamer expertise on their purchase intention.

*H5b:* Viewers’ flow experience mediates the effect of streamer expertise on their follow intention.

*H5c:* Viewers’ flow experience mediates the effect of streamer entertainment on their purchase intention.

*H5d:* Viewers’ flow experience mediates the effect of streamer entertainment on their follow intention.

### 3.6 The moderating effect of OSL

The consumers’ OSL is consistently associated with consumer behaviors driven by curiosity, the desire for variety, and willingness to take risks (Steenkamp and Baumgartner, 1992). People with high OSL need novel and exciting environments to experience psychological pleasure, as those with a lower-level look for familiarity and comfort (Zheng et al., 2023). Richard and Chebat (2016) also suggested that people with a high OSL tend to seek more intricate and new experiences in online realms; hence, they can influence their information processing, emotional reactions, as well as make some decisions on purchase. This means that viewers with higher levels are likely to consider streamers who have greater expertise and entertainment as trusted, because these skills embody their need for more exciting and interactive data. Conversely, individuals with lower OSL may not place as much value on these attributes, which can influence their perception of trust in the streamer. Flow is more likely to be experienced with expert or highly entertaining streamers by viewers who have a high OSL in the sense that they seek new and stimulating environments for psychological pleasure; thus, increasing their live streaming experience. Individuals with a lower OSL, preferring familiarity and comfort, might not respond as strongly to these streamer attributes. Thus, this study proposes the following hypotheses.

*H6a:* Viewers’ OSL positively moderates the impact of streamer expertise on their trust.

*H6b:* Viewers’ OSL positively moderates the impact of streamer entertainment on their trust.

*H6c:* Viewers’ OSL positively moderates the impact of streamer expertise on their flow experience.

*H6d:* Viewers’ OSL positively moderates the impact of streamer entertainment on their flow experience.

**Figure 1:**
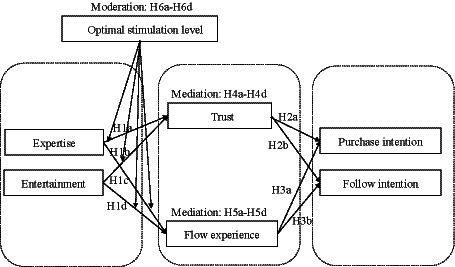
Research model.

According to the reasoning above, Figure 1 shows the research model of this study.

## 4 Research methodology

### 4.1 Measurement

This study utilized a questionnaire survey approach. The questionnaire consists of two sections: the initial section collects demographic data, while the subsequent section evaluates the research variables. These variables include independent variables (streamer expertise, entertainment), mediating variables (consumers’ trust, flow experience), dependent variables (purchase intention and follow intention), and the moderating variable (optimal stimulation level). Additionally, an attention check question is included, requiring a specific response to filter out non-serious responses.

The questionnaire utilizes established scales from previous research for all variables. Streamer expertise is gauged using four items, with two items sourced from Zheng et al. (2023) and the other two from Guo et al. (2022). The assessment of entertainment is based on four items derived from the studies conducted by Liu et al. (2022). Trust comprises three items from Shang et al. (2023). Flow experience is evaluated with four items from Chen and Lin (2018). Purchase intention is assessed using three measures derived from Dodds et al. (1991). The questionnaire items for “follow intention” were adapted from Casaló et al. (2017) and subsequently modified to align with the context of live streaming scenarios. Lastly, the OSL is determined using seven items from Zheng et al. (2023). Details of these scales are listed in Table 1.

**Table 1:** Measurement items for the variables.

Latents	Constructs	Items	References
Expertise	EX1	“The streamer possesses professional skills.”	Zheng et al. (2023) and Guo et al. (2022)
	EX2	“The streamer has special expertise.”	
	EX3	“The streamer is highly knowledgeable about products he/she promotes.”	
	EX4	“This streamer is highly experienced in live streaming and sales.”	
Entertainment	EN1	“Watching this live streaming was entertaining.”	Liu et al. (2022)
	EN2	“Watching this live streaming relaxed me.”	
	EN3	“Watching this live streaming gave me pleasure.”	
	EN4	“This live streaming that I watched was imaginative.”	
Trust	PT1	“I think that the product information of this live streaming is trustworthy.”	Shang et al. (2023)
	PT2	“I think that the streamer on this live streaming is reliable.”	
	PT3	“I anticipate that the things I purchase from this live streaming platform will meet my expectations.”	
Flow experience	FLOW1	“I was highly attentive (immersed) while watching this live streaming.”	Chen and Lin (2018)
	FLOW2	“I forgot the passage of time while watching this live streaming.”	
	FLOW3	“I was able to concentrate without being distracted while watching this live streaming.”	
	FLOW4	“Watching this live streaming took me away from the real world for a while.”	
Optimal stimulation level	OSL1	“Instead of trying something new or different, I prefer to do something familiar.”	Zheng et al. (2023)
	OSL2	“I like to experience novelty and change in my daily life.”	
	OSL3	“I like a job that brings variety, even with some dangers.”	
	OSL4	“I prefer a fixed lifestyle to one that is unpredictable.”	
	OSL5	“I’m always looking for new ideas and experiences.”	
	OSL6	“I like changing activities.”	
	OSL7	“I like to look for new and unfamiliar experiences when things get boring.”	
Follow intention	POST1	“I have the intention to watch this streamer’s live stream in the near future.”	Casaló et al. (2017)
	POST2	“I predict that I will follow this streamer.”	
	POST3	“I will actively seek out other live programs hosted by this streamer.”	
Purchase intention	PUR1	“I am highly likely to purchase the products recommended by this streamer.”	Dodds et al. (1991)
	PUR2	“I am willing to buy the products recommended by this streamer.”	
	PUR3	“I would recommend the products recommended by this streamer to others.”	

### 4.2 Data gathering and sample selection

The data collection process was specifically designed to target a segment of the population that closely mirrors the primary user base of China’s e-commerce live streaming market. The sample comprised undergraduate students under the authors’ instruction, as well as postgraduate, doctoral, and MBA students from their colleagues’ classes. This deliberate selection of participants across various educational stages, predominantly aged between 20 and 40 years, ensured an adequate representation of the demographic that dominates the live streaming consumer market in China (iiMedia Research, 2022).

For the data gathering phase, participants were instructed to utilize their mobile devices to randomly access and engage with a live streaming session on a selected platform. This engagement lasted for a duration of 10 min, post which, the participants were required to complete a survey. The questionnaire was administered through Wenjuanxing,^1^ a widely used and reputable online platform in China for academic and market research data collection. Initially, a total of 406 questionnaires were collected. However, after a thorough data cleansing process, which involved removing responses due to insufficient answering time and failure to pass the attention check question, 7 questionnaires were discarded. This resulted in 399 valid responses for our analysis. Detailed demographic information of the respondents from the 399 valid questionnaires collected is presented in Table 2. The gender distribution of the sample skews slightly female, with 53.9% of respondents identifying as female and 46.1% as male. Age-wise, the majority of the sample falls within the 20–30 age range, representing 55.9%, followed by the 31–40 age range at 41.6%. Those in the 41–50 age bracket account for a smaller portion at 2.5%, with no participants recorded above 50 years of age. The age distribution of the respondents aligns with the mainstream user base of China’s e-commerce live streaming market, indicating that the sample is representative of the target demographic. The average weekly e-commerce live streaming viewing duration varied, with the most substantial proportion of the sample, 44.9%, watching 1–3 h. The next largest group, at 26.1%, reported viewing 3–7 h weekly. Fewer respondents, 19.8%, watch for less than 1 h, and a minority of 9.3% watch for more than 7 h. Regarding the types of products featured in the live stream that participants had just viewed prior to completing the survey, search products were predominant, comprising 47.9% of responses. Experience products were next, at 29.3%, followed by credence products at 22.8%.

**Table 2:** Demographic characteristics.

Index	Category	Frequency	Percent
Gender	Male	184	46.1%
	Female	215	53.9%
Age	20–30	223	55.9%
	31–40	166	41.6%
	41–50	10	2.5%
	Above 50	0	0%
Average weekly hours spent watching live e-commerce	Less than 1 h	79	19.8%
	1–3 h	179	44.9%
	3–7 h	104	26.1%
	Above 7 h	37	9.3%
Product type from just-watched live stream	Search products	191	47.9%
	Experience products	117	29.3%
	Credence products	91	22.8%

## 5 Data analyses and results

### 5.1 Common method bias

Because the data for multiple variables in this study were collected from the same group of participants and primarily through questionnaire surveys, it raises the possibility of common method bias. To assess this possibility, we employed Harman’s single-factor test based on both confirmatory factor analysis (CFA) and exploratory factor analysis (EFA). The specific approach of Harman’s single factor test based on CFA involves loading all measurement items of the study variables onto one common factor, thus constructing a single-factor structural equation model, and then evaluating the fit of this model. The results indicate that the fit of this single-factor model is not ideal, with χ^2^/df = 7.865, RMSEA = 0.131, NFI = 0.553, CFI = 0.584, and IFI = 0.586, suggesting to a certain extent that there is no serious common method bias in this study.

This study employed Harman’s single-factor test, which is based on exploratory factor analysis (EFA), to further evaluate the presence of probable common method bias in our dataset. This test assumes that if a single factor emerges or one general factor accounts for the majority of covariance among variables, common method variance might be present. The greater the explained variance by this factor, the more pronounced the bias. If the variation of a single factor is 40% or higher under criteria put forth by Podsakoff and Organ (1986), this suggests significant common method bias. Using SPSS 26.0, we carried out an EFA in our collected data without rotation of factors. The explained variance for the first common factor amounted only to 35.04%, which is below threshold, indicating that common method bias is not a major problem in our study.

### 5.2 Reliability and validity testing

Prior to conducting hypothesis testing on the research model, we conducted assessments on the scales used in this study to determine their reliability and validity. Initially, we assessed the internal consistency of the measures by analyzing the factor loadings of the items on their respective constructs and the values of Cronbach’s α coefficient. Table 3 shows that all factor loadings above the minimum threshold of 0.5, indicating a robust correspondence between the items and their intended constructs. Furthermore, the Cronbach’s α coefficient values for all constructs above the minimum acceptable level of 0.70, demonstrating the strong internal consistency and reliability of our questionnaire.

**Table 3:** Reliability and validity.

Variables	Constructs	Standard loading	Cronbach’s α	CR	AVE
Expertise	EX1	0.783	0.858	0.858	0.602
	EX2	0.769			
	EX3	0.761			
	EX4	0.789			
Entertainment	EN1	0.789	0.860	0.860	0.606
	EN2	0.766			
	EN3	0.781			
	EN4	0.777			
Trust	PT1	0.730	0.817	0.819	0.602
	PT2	0.803			
	PT3	0.793			
Flow experience	FLOW1	0.793	0.863	0.864	0.614
	FLOW2	0.769			
	FLOW3	0.818			
	FLOW4	0.751			
Optimal stimulation level	OSL1	0.791	0.917	0.917	0.612
	OSL2	0.784			
	OSL3	0.782			
	OSL4	0.801			
	OSL5	0.774			
	OSL6	0.772			
	OSL7	0.771			
Follow intention	POST1	0.798	0.810	0.811	0.589
	POST2	0.754			
	POST3	0.749			
Purchase intention	PUR1	0.822	0.844	0.844	0.644
	PUR2	0.797			
	PUR3	0.787			

To evaluate the validity of our measurement model, this study performed a validation check using AMOS 24.0, examining both convergent and discriminant validity. The findings in Table 3 demonstrate that the composite reliability (CR) values ranged from 0.811 to 0.917 for all variables, surpassing the acceptable threshold of 0.7 (Hair et al., 2013). Additionally, the average variance extracted (AVE) for each variable ranged from 0.589 to 0.644, exceeding the minimum threshold of 0.5 (Fornell and Larcker, 1981), confirming convergent validity. Furthermore, the results presented in Table 4 indicate that the square roots of the AVE for each construct are higher than the correlation coefficients with other constructs. This suggests that there is sufficient evidence to establish discriminant validity, in accordance with the criterion proposed by Fornell and Larcker (1981).

**Table 4:** Discriminant validity.

Latent variance	Mean	SD	1	2	3	4	5	6	7
Expertise	3.308	0.966	**0.776**						
Entertainment	3.314	0.971	0.492***	**0.778**					
Trust	3.378	0.983	0.438***	0.472***	**0.776**				
Flow experience	3.313	0.975	0.433***	0.458***	0.502***	**0.783**			
Optimal stimulation level	3.346	0.962	0.482***	0.495***	0.436***	0.424***	**0.782**		
Follow intention	3.346	0.989	0.403***	0.496***	0.411***	0.367***	0.453***	**0.768**	
Purchase intention	3.269	1.072	0.398***	0.486***	0.483***	0.461***	0.538***	0.498***	**0.802**

***Means *p* < 0.001. The bold numbers on the diagonal are the square root of the AVE of the corresponding variable.

### 5.3 Hypothesis testing

**Figure 2:**
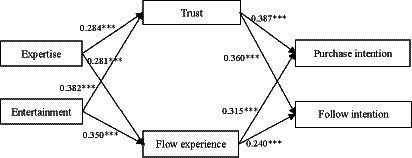
Structural model results. ***Means *p* < 0.001.

The hypotheses were tested using a structural equation model, the results of which are displayed in Figure 2. The model demonstrates an excellent overall fit, as indicated by the following statistics: *χ*^2^/df = 1.479, SRMR = 0.061, RMSEA = 0.035, GFI = 0.938, AGFI = 0.920, IFI = 0.978, TLI = 0.974, CFI = 0.978. The results of the path coefficients and their respective significance levels are presented in Table 5. The findings demonstrate the following: streamer expertise positively influenced viewers’ trust (*β* = 0.284, *p* < 0.001) and flow experience (*β* = 0.281, *p* < 0.001), supporting H1a and H1b. Similarly, streamer entertainment also positively influenced viewers’ trust (*β* = 0.382, *p* < 0.001) and flow experience (*β* = 0.350, *p* < 0.001), supporting H1c and H1d. Furthermore, the results reveal that viewers’ trust had a positive impact on their purchase intention (*β* = 0.387, *p* < 0.001) as well as their follow intention (*β* = 0.360, *p* < 0.001), supporting H2a and H2b. Lastly, viewer’s flow experience is positively associated with their purchase intention (*β* = 0.315, *p* < 0.001) and follow intention (*β* = 0.240, *p* < 0.001), thus confirming H3a and H3b.

**Table 5:** Path analysis.

Hypothesis	Path	Std. Estimate	Estimate (β)	S.E.	C.R.	*p*-value	Result
H1a	EX → PT	0.258	0.284	0.059	4.395	***	Support
H1b	EX→ FLOW	0.272	0.281	0.061	4.438	***	Support
H1c	EN → PT	0.356	0.382	0.062	5.722	***	Support
H1d	EN → FLOW	0.347	0.350	0.064	5.421	***	Support
H2a	PT → PUR	0.471	0.387	0.074	6.342	***	Support
H2b	PT → POST	0.400	0.360	0.071	5.616	***	Support
H3a	FLOW→PUR	0.360	0.315	0.066	5.442	***	Support
H3b	FLOW→POST	0.251	0.240	0.063	3.987	***	Support

***Means *p* < 0.001. EX, expertise; EN, entertainment; PT, trust; FLOW, flow experience; PUR, purchase intention; POST, follow intention.

### 5.4 Mediation effect test

This study conducted mediation analyses using the Bootstrap method with 5,000 resamples via Bootstrap ML estimation in AMOS, as detailed in Table 6. The indirect effects of streamer expertise on viewers’ purchase intention (*β* = 0.110) and their follow intention (*β* = 0.102) via trust were significant, with *p*-values well below 0.001 and the 95% confidence intervals for these effects did not include zero, substantiating the mediating role of trust, in line with hypotheses H4a and H4b. Similarly, the indirect effects of streamer entertainment on viewers’ purchase intention (*β* = 0.148) and their follow intention (*β* = 0.137) via trust were also significant, with *p*-values well below 0.001 and the 95% confidence intervals did not include zero, supporting H4c and H4d.

**Table 6:** The results of mediation test.

Hypothesis	Path	Effect size (β)	S.E.	*p*-value	Bootstrap 95%CI		Result
					LLCI	ULCI	
H4a	EX→PT → PUR	0.110	0.032	***	0.054	0.179	Support
H4b	EX→PT → POST	0.102	0.031	***	0.051	0.174	Support
H4c	EN → PT → PUR	0.148	0.036	***	0.083	0.224	Support
H4d	EN → PT → POST	0.137	0.038	***	0.076	0.224	Support
H5a	EX→FLOW→PUR	0.089	0.028	0.001	0.040	0.147	Support
H5b	EX→FLOW→POST	0.068	0.026	0.002	0.026	0.131	Support
H5c	EN → FLOW→PUR	0.110	0.032	***	0.052	0.179	Support
H5d	EN → FLOW→POST	0.084	0.030	0.002	0.035	0.155	Support

***Means *p* < 0.001. EX, expertise; EN, entertainment; PT, trust; FLOW, flow experience; PUR, purchase intention; POST, follow intention.

Furthermore, streamer expertise demonstrated a significant indirect effect through flow experience on purchase intention, exhibiting a magnitude of impact of 0.089, and on follow intention with an effect size of 0.068. Although these mediating effects are relatively smaller, they are statistically significant with p-values of 0.001 and 0.002, respectively, and the 95% confidence intervals are entirely above zero, indicating reliable mediation, supporting H5a and H5b. Similarly, entertainment through flow experience had a mediating effect on purchase intention at 0.110 and on follow intention at 0.084. These results suggest that entertaining content enhances viewers’ flow experience, which in turn increases their intentions to purchase and to follow post-viewing. The statistical significance of these mediating effects is confirmed with p-values well below 0.001, and non-zero 95% confidence intervals, highlighting the importance of flow experience in the content consumption process, supporting H5c and H5d.

### 5.5 Moderation effect test

This study conducted regression analyses using PROCESS macro for SPSS version 4.2 to test for moderation effects of optimal stimulation level. To eliminate issues with multicollinearity, a centralization process was applied to the variables of streamer expertise, entertainment, and optimal stimulation level, including their respective interaction terms. The findings of moderation analysis are presented in Table 7. In Model 1, where trust serves as the outcome, streamer expertise and optimal stimulation level were both significant predictors, with coefficients of 0.237 (*p* < 0.001) and 0.249 (*p* < 0.001), respectively. The interaction term (EX*OSL) also yielded a significant effect with a coefficient of 0.173 (*p* < 0.001), suggesting that the relationship between streamer expertise and trust is moderated by the optimal stimulation level, supporting H6a. Model 2 presents the effects on trust with entertainment as the predictor, showing a significant main effect (Coeff. = 0.240, *p* < 0.001) and a significant interaction with optimal stimulation level (EN*OSL) (Coeff. = 0.230, *p* < 0.001). This indicates that viewers’ OSL also moderates the impact of entertainment on trust, supporting H6b.

**Table 7:** The results of moderation analysis.

Antecedents	Consequent: PT						Consequent: FLOW					
	Model 1			Model 2			Model 3			Model 4		
	Coeff.	SE	*p*-value	Coeff.	SE	*p*-value	Coeff.	SE	*p*-value	Coeff.	SE	*p*-value
Constant	3.309	0.048	***	3.283	0.047	***	3.227	0.047	***	3.222	0.047	***
EX	0.237	0.050	***	—	—	—	0.231	0.049	***	—	—	—
EN	—	—	—	0.240	0.050	***	—	—	—	0.245	0.049	***
OSL	0.249	0.051	***	0.240	0.050	***	0.231	0.050	***	0.232	0.050	***
EX*OSL	0.173	0.050	***	—	—	—	0.217	0.049	***	—	—	—
EN*OSL	—	—	—	0.230	0.047	***	—	—	—	0.220	0.046	***
	$R^{2}=0.$ 226 $\Delta R^{2}=0.024$ *p* < 0.001			$R^{2}=0.$ 258 $\Delta R^{2}=0.045$ *p* < 0.001			$R^{2}=0.$ 238 $\Delta R^{2}=0.038$ *p* < 0.001			$R^{2}=0.$ 253 $\Delta R^{2}=0.042$ *p* < 0.001		

*** Means *p* < 0.001. EX, expertise; EN, entertainment; PT, trust; FLOW, flow experience; OSL, optimal stimulation level.

In the context of flow experience, Model 3 demonstrates that streamer expertise has a significant main effect (Coeff. = 0.231, *p* < 0.001). The interaction of expertise with optimal stimulation level (EX*OSL) again is significant (Coeff. = 0.217, *p* < 0.001), reinforcing the moderating role of OSL in the expertise-flow relationship, supporting H6c. Finally, Model 4, which predicts flow experience based on entertainment, reveals a significant main effect (Coeff. = 0.245, *p* < 0.001) and a significant interaction effect (EN*OSL) (Coeff. = 0.220, *p* < 0.001). This pattern mirrors the findings for trust, underscoring the consistent moderating influence of viewers’ OSL on the relationship between entertainment and flow experience, supporting H6d.

**Figure 3:**
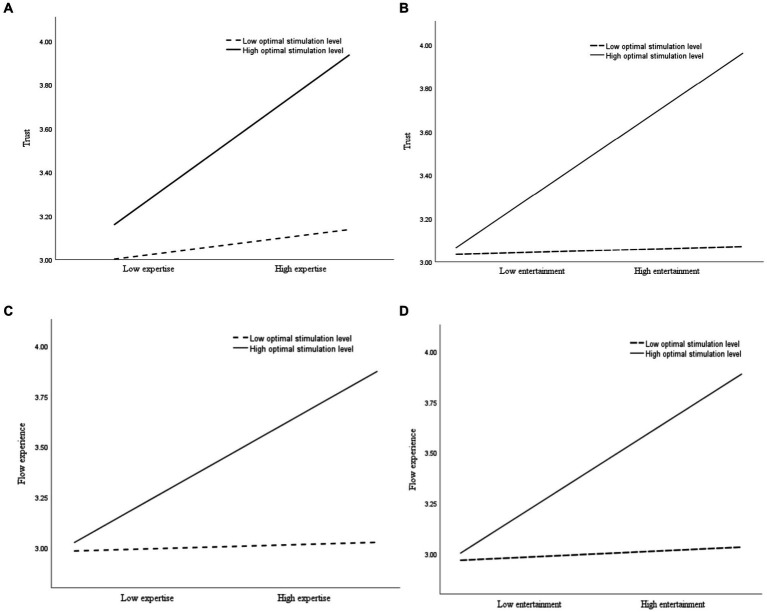
**(A–D)** Moderation effect of optimal stimulation level.

In order to visually demonstrate the moderating impact of OSL, this study categorized the participants into two groups based on the standard deviation criteria (+1SD/-1SD) and created simple effects graphs. According to Figure 3, for trust as the outcome variable, the effect of streamer expertise at low optimal stimulation levels was not significant, as the relationship depicted by the dashed line in Figure 3A is relatively flat. However, at high OSL, there was a strong and positive correlation between expertise and trust, with the solid line indicating a steeper incline. This suggests that for viewers with a high OSL, increases in streamer expertise are associated with greater increases in trust.

Similarly, the effect of entertainment on trust at low OSL was again non-significant, as shown by the flat dashed line in Figure 3B. Yet, at high OSL, entertainment’s impact on trust became significantly positive, indicated by the upward sloping solid line. This pattern denotes that consumers with a higher OSL may perceive more trust as entertainment value increases.

Regarding flow experience, a similar pattern emerged. Figure 3C demonstrates that at low levels of optimal stimulation, the expertise’s effect on flow experience was minimal. In contrast, at high OSL, expertise had a significant positive impact on flow experience, as seen by the steeper slope of the solid line. Figure 3D shows that for entertainment, the slope was again flat at low OSL, indicating a minimal effect on flow experience. At high OSL, the positive relationship between entertainment and flow experience became pronounced, as evidenced by the upward trajectory of the solid line.

These findings suggest that viewers’ OSL significantly moderates the effects of streamer expertise and entertainment on their trust and flow experience. Specifically, the effects of streamer expertise and entertainment are amplified when viewers’ OSL are high, leading to higher levels of trust and flow experience.

## 6 Discussions and implications

### 6.1 Discussion

This study explores the intricate connections between streamer expertise (entertainment) and viewers’ purchase intention and follow intention. It examines the mediating roles of trust and flow experience, along with the moderating influence of OSL. The key findings are as follows:

First, consistent with the findings of Zhang et al. (2022a), this study also confirms that streamer expertise has a positive impact on viewers’ trust, suggesting that streamers’ expertise plays a crucial role in cultivating trust and credibility among consumers. This study supports the hypothesis that streamer expertise has a positive impact on viewers’ flow experience. Viewers are more likely to have an engaging experience with streamers who demonstrate high levels of expertise in the content they create. Furthermore, our study found that streamer entertainment positively affects viewers’ trust and enhances their flow experience, consistent with Liu et al. (2022)’s findings.

Second, this study further substantiates the positive effect of trust by consumers on purchase intention as established in previous literature (Park and Lin, 2020; Lu and Chen, 2021; Meng et al., 2021; Liu et al., 2022; Zhou and Tong, 2022). This research also indicates a significant relationship between viewers’ trust and follow up behavior after viewing, hence partially supporting Zhang et al.’s (2022b) findings that consumers’ perception of the reliability of streamers significantly contributes to trust in streamers, and significantly affects their intention to continue watching. In a similar vein, our findings corroborate the positive impact of viewers’ flow experience with live streaming on their purchase intention as can be observed from other consumer decision-making studies (Richard and Chebat, 2016; Sun et al., 2019; Huang et al., 2022; Liao et al., 2022; Liu et al., 2022; Saffanah et al., 2023). Moreover, a new feature of this research is that the flow experience positively influences viewers’ intent to follow up after viewing which supports Kim and Kim’s (2022) conclusion about the effect of the flow on consumer loyalty.

Finally, the results of this study indicate that optimal stimulation level moderates both streamer expertise and entertainment influence on consumers’ trust and flow experience. Specifically, at higher optimal stimulation levels, the effects of streamer expertise and entertainment are amplified, leading to increased levels of consumers’ trust and engagement.

### 6.2 Theoretical implications

This study contributes significantly to the extant literature on e-commerce live streaming and consumer behaviors in several crucial perspectives, presenting insights that are valuable theoretically.

First, the study contributes to the theoretical background on consumer behavior in live streaming commerce through introducing and underlining follow intention. The major aspect of this is longer-term relational dynamics as opposed to the typical focus on short-term purchasing and hence, a more rounded theory has been provided in relation to an area often ignored by previous studies.

Second, the theoretical contribution of this study is novel due to the application of optimal stimulation level concept in live streaming commerce. This opens up a new angle in the understanding of opportunities to effect consumer behavior using streamers, and thus presents necessary theoretical grounds for more personalized and efficient marketing strategies within this segment.

Finally, this study provides a new consumer intra-process model that combines trust and flow experience perspective for understanding the psychological processes of consumers in live streaming commerce contexts. It does not only contribute to the development of theoretical background on e-commerce and consumer’s behavior but offers great impulse for market strategy forming and interdisciplinary investigations.

### 6.3 Practical implications

First, for live streaming e-commerce platforms, the expertise and entertainment value of streamers are effective strategies to cultivate consumer trust and enhance flow experience. Platforms should prioritize selecting streamers with solid expertise and entertainment skills to boost consumer purchase intention and cultivate a loyal viewer base through fostering consumer trust and enhancing flow experience. Besides, it is important for companies to invest in significant training programs for streamers oriented toward broadening their knowledge of the subject and developing better presentation skills. For instance, workshops focused on the skill of persuasive storytelling, efficient audience engagement methods and effective utilization of visuals can greatly improve streamer ability. Besides such specialized workshops periodical training sessions regarding updated material in the industry will continue to make sure that content produced by streamers remain current as well informative.

Second, in order to guarantee the viewability of the content, it is necessary for e-commerce platforms to develop and implement algorithms that tailor live stream material based on viewers’ desired levels of stimulation. This can be determined by looking into the past number of views to understand what kind of speed, complexity and engagement they prefer in content. For example, people with the higher level of stimulating preference might be offered to follow active and instantaneous examples whereas others prefer more detailed and leisure information.

Third, to create a lasting bond with the viewers, live stream sponsors should come up with ways of interacting with their viewers after streaming. This could mean sending tailored-follow up emails with exclusive deals, organizing a Q&A session after-stream or offering more content for individuals who follow subscribe. These strategies can be efficient in turning casual viewers into loyal supporters.

Lastly, the incorporation of state-of-the-art data analytics solutions can provide a deeper dive into consumer engagement with live streams. This information can be used to improve the streaming slots, content types and modes for engaging. For example, through analytics, it is possible to determine, from among the available content segments, those that are most appealing, as well as viewing preferences and best time of streaming; such findings will be useful in refining upcoming streams.

### 6.4 Limitations and future research directions

Although this study offers significant insights into consumer behavioral intentions in live streaming commerce, it is crucial to mention its limitations. Awareness of these shortcomings can point the future direction for further research. First, the methodology involved participants only watched a 10-min live stream, and this may not capture all the consumer experiences and behaviors in a real live streaming session. Further studies could look into the influence of extending the duration on consumer perceptions and behaviors to gain a more holistic account for the live streaming experience. Second, this study relied heavily on quantitative approaches. Quantitative research often assumes that certain factors will affect consumer behavior, while ignoring other influencing factors, resulting in an incomplete understanding of the new phenomenon of live shopping. Further studies are suggested to include qualitative methods, such as interviews, in order to reveal the psychological mechanisms and phenomenology of customer engagement during live shopping. Third, in the light of progression concerning live streaming techs and changes emerging from customers’ sides it would be interesting for future research to observe how new features including such ones as VR or some elements changing interactivity influence consumers reactions. This could be significant in terms of implications regarding the role technology plays in consumer engagement and even purchase decision-making over the broadcast platforms. Lastly, this study employed non-random judgment sampling, and the selected sample’s age distribution aligns with that of typical users of livestreaming e-commerce platforms in China, to some extent enhancing the reliability and applicability of the research findings. However, it is important to note that the data for this study were limited to China, which might restrict the external validity and generalizability of the results. Future research could broaden the sources of the sample, including participants from different regions and diverse cultural backgrounds, to conduct cross-cultural comparative studies.

## Data availability statement

The original contributions presented in the study are included in the article/supplementary material, further inquiries can be directed to the corresponding authors.

## Ethics statement

Ethical review and approval was not required for the study on human participants in accordance with the local legislation and institutional requirements. Written informed consent from the [patients/participants OR patients/participants legal guardian/next of kin] was not required to participate in this study in accordance with the national legislation and the institutional requirements.

## Author contributions

YJ: Conceptualization, Data curation, Formal analysis, Methodology, Writing – original draft. H-TL: Conceptualization, Project administration, Supervision, Writing – review & editing. WL: Data curation, Investigation, Methodology, Writing – review & editing.

## Footnotes

^1^https://www.wjx.cn/
